# Circular RNAs in Plasma and Beyond: Potential Biomarkers for Breast Cancer

**DOI:** 10.32604/or.2026.085395

**Published:** 2026-09-14

**Authors:** Chunming Wang, Xu Wang, Yubo Liu, Jia Xu, Pingfa Li

**Affiliations:** School of Medical Laboratory, North Henan Medical University, Xinxiang, China

**Keywords:** Circular RNAs, plasma biomarkers, breast cancer (BC), liquid biopsy, BC management, clinical translation

## Abstract

Breast cancer (BC) continues to be a major cause of cancer-related mortality among women, and early diagnosis remains critical for improving survival outcomes. Conventional tissue biopsy and imaging techniques are constrained by invasiveness and limited sensitivity in early-stage disease, whereas routine serum tumor markers lack sufficient specificity for reliable early detection. Circular RNAs (circRNAs) have increasingly been recognized as promising non-invasive biomarkers, owing to their remarkable stability and detectability in plasma. Here, we summarize the current landscape of plasma circRNAs as diagnostic, prognostic, and chemoresistance-related biomarkers in BC, emphasizing their clinical relevance in therapy selection, subtype stratification, treatment monitoring, and recurrence prediction. Aberrant circRNA expression in the plasma of BC patients has been associated with tumor size, stage, and molecular subtype, and functionally linked to cell survival, metastasis, and drug resistance. Importantly, circRNAs may complement conventional approaches including imaging and serum markers, and their integration with other circulating analytes could enhance diagnostic precision and support individualized therapeutic decisions. Our work consolidates recent advances in circRNA research across preoperative, postoperative, drug resistance, and recurrence settings, providing a framework for non-invasive diagnosis, clinical decision-making, and outcome prediction in BC management. This review aims to bridge tissue-based discoveries and plasma-based applications for the clinical translation of circRNAs.

## 1 Introduction

Breast cancer (BC) is a heterogeneous disease originating from mammary epithelial cells [1]. Despite therapeutic progress, BC remains a leading cause of cancer-related death in women, highlighting the critical need for early diagnosis and longitudinal surveillance. Post-surgical management is guided by histopathological assessment and may involve chemotherapy, radiotherapy, endocrine therapy, targeted agents, or immunotherapy [2]. BC comprises multiple molecular subtypes with distinct biology and treatment responses, yet subtype assignment depends on tissue biopsy, which cannot be repeated over time [3]. While tissue studies have linked circular RNA (circRNA) expression to specific BC subtypes, their applicability to plasma-based subtyping warrants further investigation [4]. This need has motivated the search for circulating biomarkers that reflect tumor evolution and complement existing subtyping tools.

Current BC monitoring relies on imaging techniques and serum tumor markers, whereas tissue biopsy remains the gold standard for definitive diagnosis and subtype classification [5]. However, each of these approaches has inherent limitations. Mammography sensitivity is compromised in dense breast tissue and is associated with higher false-positive rates [6]. Tissue biopsy is invasive, precludes repeated sampling, and may miss intratumoral heterogeneity and clonal evolution [7]. Serum markers are also elevated in benign conditions and have limited diagnostic accuracy [8]. These limitations highlight the need for alternative non-invasive biomarkers. CircRNAs are being actively investigated as promising candidates for BC biomarker discovery [9].

Plasma dysregulation of circRNAs has been consistently documented in BC patients, and mechanistic evidence supports their involvement in tumor progression, metastatic dissemination, and chemoresistance through diverse molecular pathways [10]. As non-invasive biomarkers, plasma circRNAs offer several inherent advantages. Their covalently closed circular structure confers resistance to exonuclease degradation, ensuring reliable detectability in circulation. Moreover, their expression levels can be measured repeatedly serially, facilitating longitudinal monitoring of tumor dynamics [11]. In this review, we summarize the current evidence on plasma circRNAs as non-invasive biomarkers for BC, with a focus on their diagnostic, prognostic, chemoresistance-related, and recurrence prediction applications. We also aim to provide a balanced perspective on the opportunities and challenges associated with plasma circRNA biomarkers in BC management, while extending our scope to include tissue-based findings that guide clinical application.

## 2 Search Strategy

To identify relevant studies on plasma circRNAs as potential biomarkers for BC, we performed a systematic search of the PubMed database. The search strategy incorporated terms related to circRNAs (“circular rna” OR “circRNA”), specimen types (“serum” OR “plasma” OR “whole blood”), and the disease (“breast cancer” OR “BC”). We restricted the search to English-language articles published within the past ten years and excluded review articles during the initial screening. This search yielded 384 records. Two authors (C.M. Wang and X. Wang) independently evaluated titles and abstracts against predefined eligibility criteria. Studies were included if they: (1) examined circRNA expression in plasma, serum, or whole blood from breast cancer patients; (2) reported findings pertaining to diagnosis, prognosis, or chemoresistance; and (3) provided quantitative data on circRNA expression or clinical performance. Studies were excluded if they were non-original contributions such as reviews, editorials, and case reports, if they were not conducted in human samples, or if they did not specifically address breast cancer. Disagreements between authors were resolved through discussion. The search strategy was developed with reference to previously published systematic reviews on circRNA biomarkers [12] and tailored to the scope of the present review.

## 3 CircRNAs

### 3.1 Biogenesis and Classification of CircRNAs

CircRNAs were initially identified in plant viroids, viruses, and yeast. For many years, they were considered incidental products of precursor messenger RNAs (pre-mRNAs) splicing and were thought to lack biological function [13]. Growing evidence now indicates that aberrant circRNA expression contributes to the pathogenesis and progression of multiple human diseases [14,15]. Understanding their biogenesis is therefore of fundamental importance. Mechanistically, circRNAs are generated from pre-mRNAs through back-splicing, a process in which a downstream 5′ splice donor is covalently joined to an upstream 3′ splice acceptor, producing a covalently closed circular transcript, in contrast to the sequential 5′-to-3′ exon joining of canonical linear splicing [16].

**Figure 1:**
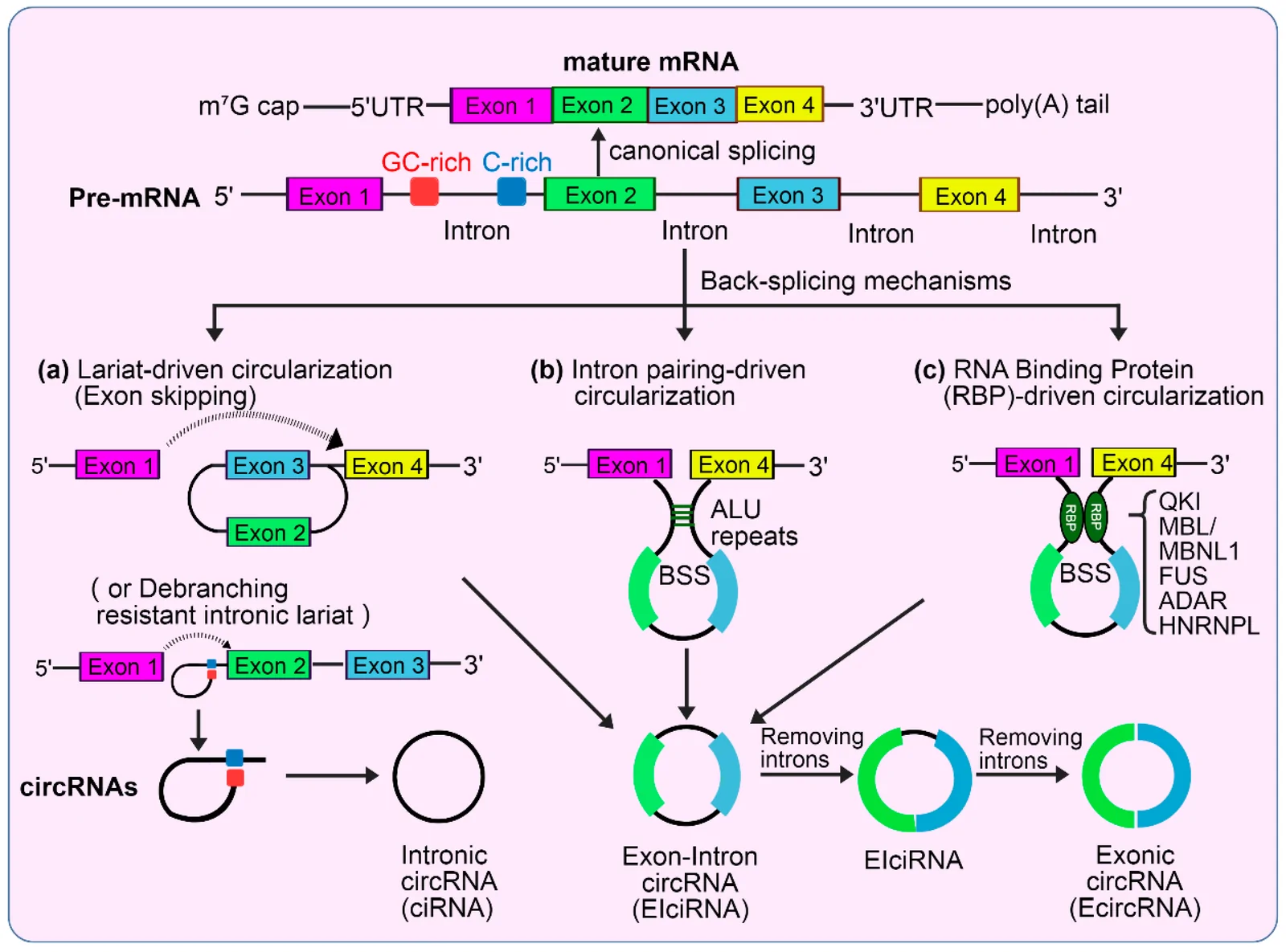
**Mechanisms of circular RNA (circRNA) biogenesis and classification.** Schematic illustration of the three major back-splicing mechanisms: (**a**) lariat-driven circularization (exon skipping), (**b**) intron pairing-driven circularization (mediated by complementary sequences such as Alu sequence repeats), and (**c**) RNA-binding protein (RBP)-driven circularization (facilitated by RBPs including Quaking, Muscleblind, and Muscleblind-like splicing regulator 1). CircRNAs are classified into intronic circRNAs (ciRNAs), exon-intron circRNAs (EIciRNAs), and exonic circRNAs (EcircRNAs) according to their composition. Abbreviations: ADAR, Adenosine deaminase acting on RNA; Alu, Alu sequence; BSS, Back-splice site; CircRNA, Circular RNA; CiRNAs, Circular intronic RNAs; EcircRNAs, Exonic circRNAs; EIciRNAs, Exon-intron circRNAs; FUS, Fused in sarcoma; HNRNPL, Heterogeneous nuclear ribonucleoprotein L; MBL, Muscleblind; MBNL1, Muscleblind-like splicing regulator 1; mRNA, Messenger RNA; QKI, Quaking; RBP, RNA-binding protein; UTR, Untranslated region. Image created with BioGDP.com.

CircRNA biogenesis is regulated by multiple factors, including intronic complementary sequences (ICSs), RNA-binding proteins (RBPs), and splicing factors. ICSs are short complementary elements situated within the introns flanking the exons that give rise to circRNAs; these sequences promote the pairing of upstream and downstream introns, thereby facilitating back-splicing [17]. In addition to ICSs, RBPs constitute another critical layer of regulation. Numerous RBPs, including Quaking and Muscleblind-like proteins, associate with pre-mRNA and modulate back-splicing events. Splicing factors also affect circRNA production by modulating splice site selection [18]. Collectively, these regulatory mechanisms underlie three principal models of back-splicing (Fig. 1) [19].

Based on their composition, circRNAs are broadly classified into three categories: exonic circRNAs (EcircRNAs), circular intronic RNAs (ciRNAs), and exon-intron circRNAs (EIciRNAs) [20]. EcircRNAs, composed entirely of exons, are the most abundant circRNA subtype and are predominantly localized in the cytoplasm. ciRNAs are derived from intronic lariat intermediates that escape debranching and are primarily localized in the nucleus. EIciRNAs, which contain both exonic and intronic sequences, are also predominantly nuclear and have been implicated in the regulation of gene transcription [21].

### 3.2 Characteristics of CircRNAs in Liquid Biopsy

In recent years, liquid biopsy has emerged as a transformative tool in oncology, enabling the non-invasive analysis of circulating biomarkers [22]. Among these, circRNAs have garnered increasing attention due to their distinctive biological properties and clinical potential [23]. Unlike linear RNAs, circRNAs feature a covalently closed loop structure that confers exceptional resistance to exonuclease degradation, ensuring their remarkable stability in biological fluids [24]. This inherent stability positions them as promising candidates for disease detection and monitoring.

The remarkable stability of circRNAs is further demonstrated by their resistance to various storage and processing conditions. Research has shown that circRNAs remain detectable in plasma after extended storage at −80°C, repeated freeze-thaw cycles, and even room-temperature incubation for up to 24 h [25]. Although widely expressed, their levels differ considerably across tissues and cell types. Certain circRNAs that are enriched in breast tissue are also markedly elevated in BC cells compared with normal breast epithelial cells [26]. This tissue-specific expression enables circRNAs to distinguish tumor from normal tissue, supporting their potential use as circulating biomarkers upon release into the bloodstream.

Tumor cells actively shed circRNAs into the circulation through encapsulation within exosomes or microvesicles, or via association with proteins, resulting in their widespread distribution in peripheral blood [27]. BC patients exhibit dysregulated plasma circRNA profiles compared with healthy controls, with specific circRNAs being either upregulated or downregulated depending on the circRNA species, as measured by hybridization-based assays and nucleic acid amplification. Moreover, circRNA expression profiles change dynamically over the course of clinical disease [28]. For instance, their levels decrease substantially following effective treatment and increase again upon recurrence or progression [29]. This dynamic nature makes circRNAs well-suited for longitudinal monitoring of treatment response and disease relapse in BC. Collectively, these properties, including high stability, tissue specificity, abundant presence in biofluids, and dynamic expressional changes, position circRNAs as promising non-invasive biomarkers for BC diagnosis, prognosis, and therapeutic surveillance.

### 3.3 Detection Methods of CircRNAs

Accurate detection of circRNAs is essential for their clinical translation as tumor biomarkers. Advances in molecular technologies have enabled the identification of numerous circRNAs in BC tissues, plasma, and cells. To date, however, no consensus exists regarding a superior detection method. The optimal approach should be selected based on the research objectives, sample types, and available laboratory resources [30]. Detection methods can be broadly classified into three categories according to their primary application stage: discovery, validation, and clinical quantification [31]. Discovery methods enable unbiased identification of circRNAs; validation methods serve to confirm circRNA existence and circularity; and clinical quantification methods are optimized for sensitive and specific measurement of circRNA levels in clinical samples (Table 1).

**Table 1:** Detection Methods of circular RNAs (circRNAs).

Application Stage	Methods	Key Features	Limitations	References
Discovery	Microarray	High-throughput screening of known circRNAs; relatively low cost	Lower specificity; cannot detect novel circRNAs	[32]
Discovery	RNA-Seq (short-read)	High-throughput; detects known and novel circRNAs; provides full sequence information	High cost; complex data analysis; potential for false-positive results due to ambiguous read alignment; cannot resolve full-length isoforms	[33]
Discovery	RNA-Seq (long-read; Nanopore/PacBio)	Full-length circRNA sequencing; resolves isoform complexity	High cost; lower throughput; higher error rate; emerging technology	[34]
Validation	Northern blotting	Widely used reference method for validating circRNA existence and size; visualizes isoforms	Low throughput; time-consuming; requires abundant RNA	[35]
Validation	RT-qPCR with divergent primers	Confirms back-splice junction; widely used; relatively low cost	Moderate throughput; requires careful primer design	[36]
Validation	RNase R + qPCR	Validates circularity by digesting linear RNAs	RNase R efficiency varies; may miss some circRNAs	[37]
Clinical Quantification	RT-qPCR	Reference method for clinical measurement; high specificity; well-established protocols	Low throughput; detects only one or a few circRNAs per reaction	[38]
Clinical Quantification	ddPCR	Absolute quantification; high sensitivity; no standard curve needed	Higher cost; lower throughput than RT-qPCR	[39]
Clinical Quantification	nanoString	No reverse transcription step; directly detects BSJ sequences	Equipment-dependent; relatively high cost	[40]
Clinical Quantification	Isothermal amplification (RCA, LAMP)	High sensitivity; rapid; no thermal cycler needed; suitable for point-of-care	Emerging technology; potential false-positive results	[41]
Clinical Quantification	CRISPR-Cas13-based detection	Ultrasensitive; directly recognizes BSJ; point-of-care potential	Emerging technology; specificity needs further evaluation	[42]
Clinical Quantification	Biosensors/aptasensors	Label-free detection; single-cell level; promising for clinical use	Emerging technology; requires large-scale clinical validation	[43]

Note: Aptasensors, Aptamer-Based Biosensors; Biosensors, Biosensor-Based Detection Platforms; BSJ, Back-Spliced Junction; circRNAs, Circular RNAs; ddPCR, Droplet Digital Polymerase Chain Reaction; LAMP, Loop-Mediated Isothermal Amplification; Nanopore, Nanopore Sequencing; nanoString, NanoString nCounter Analysis System; Northern blotting, Northern Blot Hybridization; PacBio, Pacific Biosciences Single-Molecule Real-Time Sequencing; qPCR, Quantitative Real-Time Polymerase Chain Reaction; RNA-Seq (long-read), Long-Read RNA Sequencing; RNA-Seq (short-read), Short-Read RNA Sequencing; RT-qPCR, Reverse-Transcription Quantitative Real-Time Polymerase Chain Reaction; RNase R + qPCR, Ribonuclease R Treatment Combined with Quantitative PCR; RCA, Rolling Circle Amplification. RT-qPCR appears in both categories due to its distinct applications: validation of BSJ using divergent primers and clinical quantification using standard protocols.

## 4 CircRNAs in BC: Detection Considerations and Molecular Mechanisms

### 4.1 Detection Considerations for CircRNAs in BC

The choice of biofluid is a critical factor influencing circRNA detection efficiency. Plasma and serum are the primary choices for circRNA analysis, with standard protocols typically requiring approximately 200 μL of blood, although some commercial platforms recommend up to 20 mL of whole blood (two 10-mL Streck tubes) to ensure sufficient plasma yield [44]. Plasma is generally preferred over serum, as the latter exhibits higher RNase activity that may facilitate circRNA degradation [44]. Saliva and urine have also been investigated as potential circRNA sources, though their circRNA content is considerably lower than that of serum, necessitating more sensitive detection approaches [45]. Of note, the required volume is assay-dependent; for cell-free RNA analysis, successful sequencing has been achieved using as little as 1.5 mL of plasma, and exosomal RNA detection has been validated with volumes as low as 50 μL of plasma [46]. For plasma collection, ethylenediaminetetraacetic acid (EDTA) is the recommended anticoagulant, whereas heparin should be avoided because it inhibits polymerase chain reaction (PCR) amplification [47]. Additionally, collection tubes must be sterile and free of nucleases to maintain RNA integrity and ensure assay reliability.

In addition to biofluid selection, the choice of detection method also plays a critical role in circRNA screening efficiency. In the majority of studies retrieved, the screening workflow began with microarray analysis of tumor tissues from BC patients to identify differentially expressed circRNAs. The most significantly dysregulated candidates were subsequently selected for validation in patient serum, typically using reverse-transcription quantitative real-time polymerase chain reaction (RT-qPCR) due to its high sensitivity and specificity [48]. Although RNA Sequencing (RNA-seq) and microarray offer high-throughput capabilities, their specificity is relatively limited and they are susceptible to false-positive results [49]. Therefore, standardization of reagents and optimization of detection protocols are critical for improving circRNA screening efficiency.

Moreover, the presence of other pathological conditions may confound circRNA expression profiles, potentially yielding false-positive signals [50]. Notably, hsa_circ_0001944 (circBCBM1, breast cancer brain metastasis 1) is upregulated not only in BC [51] but also in gastric cancer [52], and leukemia [53], indicating that it lacks cancer-type specificity. Therefore, when applying circRNAs for BC screening, it is essential to either exclude interference from benign or unrelated diseases, or to establish diagnostic panels capable of distinguishing among multiple pathological states [54]. Collectively, these considerations highlight the importance of careful experimental design and data interpretation when detecting circRNAs in clinical samples.

### 4.2 Molecular Mechanisms of CircRNAs in BC

**Figure 2:**
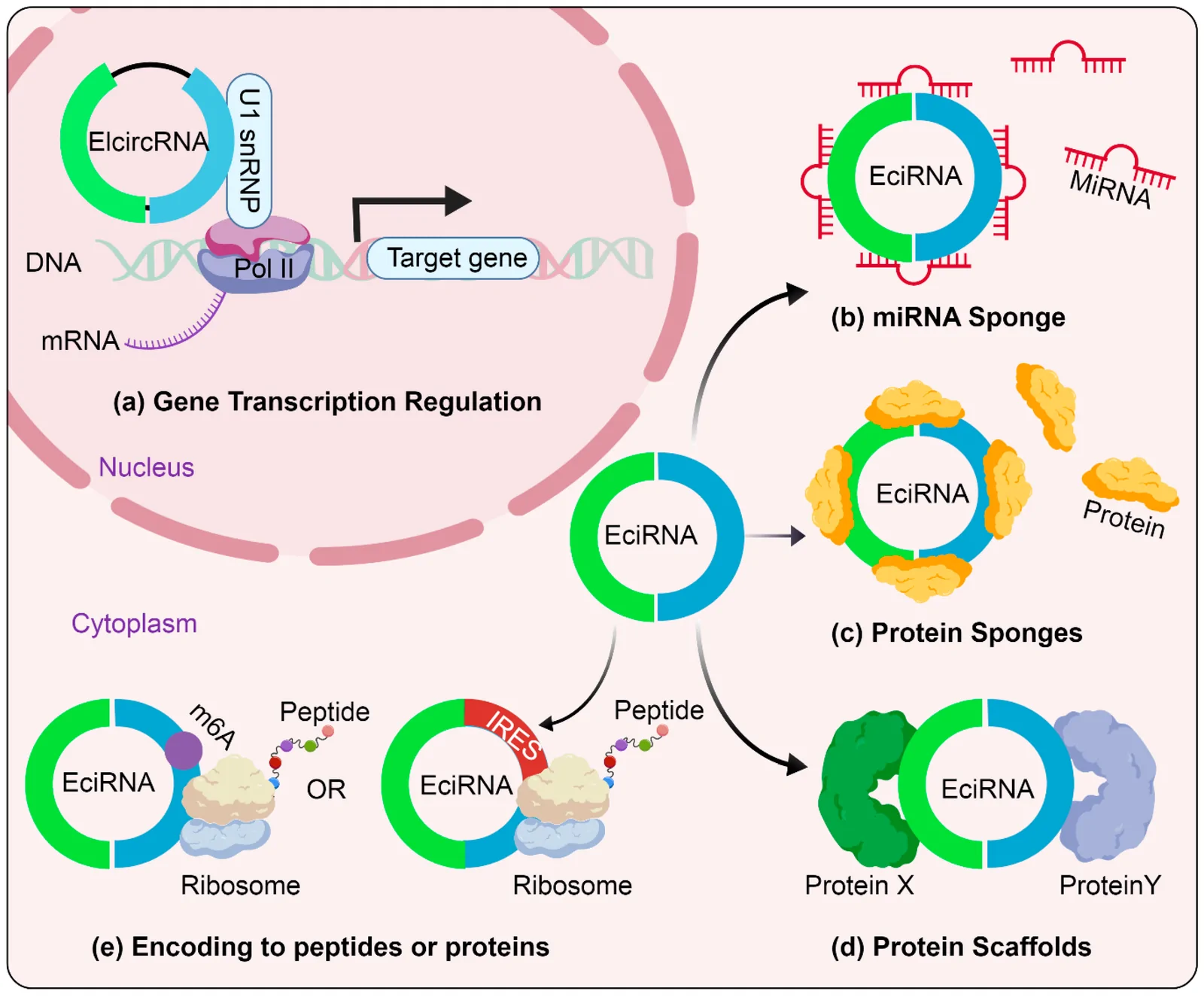
**Major molecular mechanisms of circular RNAs (circRNAs) in breast cancer (BC)**. (**a**) Transcriptional regulation: Nuclear Exon-intron circular RNAs (EIciRNAs) modulate parental gene transcription via U1 small nuclear ribonucleoprotein (U1 snRNP) or RNA polymerase II (RNA Pol II). (**b**) microRNA (miRNA) sponges: CircRNAs adsorb miRNAs to relieve suppression of target mRNAs. (**c**) Protein sponges: CircRNAs bind and sequester proteins, modulating their availability. (**d**) Protein scaffolds: CircRNAs assemble multiple proteins to facilitate complex formation and signaling. (**e**) Translation: CircRNAs encode functional peptides or proteins via internal ribosome entry sites (IRES) or N^6^-methyladenosine (m^6^A) modifications. Abbreviations: BC, Breast cancer; CircRNAs, Circular RNAs; EcircRNAs, Exonic circRNAs; EIciRNAs, Exon-intron circular RNAs; IRES, Internal ribosome entry sites; m^6^A, N^6^-methyladenosine; miRNA, microRNA; RNA Pol II, RNA polymerase II; U1 snRNP, U1 small nuclear ribonucleoprotein. Image created with BioGDP.com.

CircRNAs are extensively involved in BC progression and treatment resistance through multiple mechanisms, including microRNA (miRNA) sponging, protein binding, transcriptional regulation, and translation into functional peptides (Fig. 2) [55]. Elucidating these molecular mechanisms will help uncover novel regulatory networks in BC, provide potential targets for early screening, prognostic assessment, and real-time monitoring, and ultimately facilitate the advancement of precision medicine [56].

The competing endogenous RNA (ceRNA) mechanism, also known as the miRNA sponge, represents a core regulatory mode of circRNAs in BC. CircRNAs harbor multiple miRNA response elements (MREs) that enable them to sequester specific miRNAs, thereby preventing these miRNAs from binding to and repressing their target mRNAs [57]. Consequently, the repression of target genes is relieved, which modulates gene expression and contributes to BC progression. By sponging oncogenic or tumor-suppressive miRNAs, circRNAs fine-tune the expression of downstream target genes and subsequently regulate diverse cancer-related processes, including cell proliferation, drug resistance, metabolic reprogramming, and apoptosis [58].

Beyond miRNA sponging, circRNAs can also directly interact with proteins, functioning as decoys, scaffolds, or molecular sponges that sequester RNA-binding proteins (RBPs) [59]. Through such protein interactions, circRNAs modulate BC cell proliferation, apoptosis, cell cycle progression, and chemotherapy resistance. Specifically, circRNAs not only trap key proteins as decoys to render them functionally inactive, but also serve as scaffolding platforms that bring two or more proteins together, thereby facilitating the assembly of protein complexes and downstream signaling cascades [60]. Moreover, circRNA-protein interactions can alter the nuclear-cytoplasmic shuttling, degradation kinetics, and enzymatic activities of bound proteins. Collectively, these interactions affect the stability and function of key proteins in oncogenic pathways, including tumor protein 53 (p53), β-catenin, and epidermal growth factor receptor (EGFR) [61].

CircRNAs also influence gene expression at the transcriptional level in BC. Within the nucleus, they primarily modulate the transcription of their parental genes through interaction with U1 small nuclear ribonucleoprotein (U1 snRNP) or RNA polymerase II, thereby upregulating or downregulating the expression of oncogenes or tumor suppressors. Furthermore, by forming RNA-DNA hybrids in promoter regions, circRNAs can affect transcriptional initiation, pausing, or elongation, ultimately modulating downstream oncogenic signaling pathways [62].

In the cytoplasm, circRNAs primarily modulate protein translation through two distinct mechanisms. The first and most extensively studied mechanism involves competitive inhibition of translation, whereby circRNAs compete with linear mRNAs for ribosome or translation factor binding, thereby suppressing the translation of oncogenes or tumor suppressors [63]. The second mechanism entails the translation of circRNAs themselves into functional peptides or proteins. Certain circRNAs harbor internal ribosome entry sites (IRES) or N^6^-methyladenosine (m^6^A) modification sites, which facilitate cap-independent translation of short peptides [64].

### 4.3 Bioinformatics Resources for circRNA–miRNA Interaction Prediction

Several computational tools and databases are currently available for predicting and validating circRNA-miRNA interactions [65]. CIRcular RNA Sponge CANdidates (Cirscan), a Shiny-based web application, reconstructs circRNA-miRNA-mRNA regulatory networks from transcriptomic data and ranks candidate interactions using a sponge score. CircNet (version 2.0), an updated database for exploring circular RNA regulatory networks in cancers, offers comprehensive circRNA-miRNA-gene network data, integrating information from over 10,000 samples across 26 cancer types. The Encyclopedia of RNA Interactomes (ENCORI), also known as StarBase, provides experimentally validated miRNA-circRNA interactions derived from cross-linking and immunoprecipitation sequencing (CLIP-seq) data, serving as a useful reference for evaluating predicted targets [66]. Together, these resources can aid researchers in experimental design and in prioritizing circRNAs for further functional studies.

## 5 Classification of Blood-Based CircRNAs as BC Biomarkers

Plasma circRNAs are frequently dysregulated in BC and have garnered increasing attention as non-invasive biomarkers [66]. In this section, we summarize blood-based circRNAs, including those derived from plasma, serum, and peripheral blood, that have been reported for diagnostic, prognostic, chemoresistance-related, and recurrence-related applications in BC. To facilitate comparison, circRNAs are categorized according to their reported clinical utility; within each category, they are presented in descending order of their reported area under the curve (AUC) values. For circRNAs without AUC data, we evaluate their clinical potential based on other evidence, such as statistical significance, sample size, multi-marker panel performance, functional mechanisms, and prognostic associations. Detailed information on each circRNA, including sample type, expression direction, diagnostic performance metrics, pathway annotations, and validation status, is provided in Table 2. Importantly, the majority of the studies summarized in Table 2 did not stratify patients by molecular subtype and were conducted in unselected BC cohorts; however, a subset of studies specifically focused on triple-negative breast cancer (TNBC). This limitation is discussed in detail in Section 9.

**Table 2:** Blood-based circular RNAs (circRNAs) as biomarkers in breast cancer (BC).

CircRNA	Expression	BC Subtype	AUC	References
circRASSF2	Upregulated	Not specified	Serum: 0.9323	[67]
circCLASP1	Upregulated	Not specified	Tissue: 0.8196 Serum: 0.8902	[68]
circ-FAF1	Downregulated	Not specified	0.885	[69]
hsa_circ_0008673	Upregulated	Not specified	0.833	[70]
circRHOT1	Upregulated	Not specified	0.83	[71]
hsa_circ_0000091/circRPAP2	Upregulated	Not specified	0.974 (panel); 0.808 (with ultrasound)	[72,73]
hsa_circ_0104824	Upregulated	Not specified	0.849	[74]
circHIPK3	Upregulated	Not specified	0.8087	[75]
circ_0042881/circNF1	Upregulated	Not specified	Plasma: 0.802	[76]
hsa_circ_0001785	Upregulated	Not specified	0.784 (n = 57) 0.771 (n = 20)	[77,78]
circCDYL	Upregulated	Not specified	Serum: 0.789	[79]
circ_0065214	Upregulated	Not specified	0.78	[80]
circ0103552	Downregulated	Not specified	0.767	[81]
circABCB10/hsa_circ_0008717	Upregulated	Not specified	0.7	[81]
hsa_circ_0069094/circ_0069094	Upregulated	Not specified	0.6808	[82,83,84,85,86]
circFBXW7/hsa_circ_0001451	Downregulated	TNBC	0.655	[81]
circPRMT5	Upregulated	Not specified	NR	[87,88]
circBCBM1/hsa_circ_0001944	Upregulated	Not specified	NR	[51]
circEGFR/hsa_circ_0080222	Upregulated	TNBC	NR	[89,90,91]
circ_0000977	Downregulated	TNBC	NR	[92]
hsa_circ_0052112	Upregulated	Not specified	NR	[93]
circPSMA1	Upregulated	TNBC	NR	[94]
circSTAT3	Upregulated	TNBC	NR	[95]
circSTIL/hsa_circ_0000069	Upregulated	TNBC	0.823	[96]
circ-0001361/hsa_circRNA-0001361	Upregulated	TNBC	NR	[97]

Note: ABCB10, ATP-binding cassette subfamily B member 10; AUC, The area under the curve; BCBM1, Breast cancer brain metastasis 1; BC, Breast cancer; CDYL, Chromodomain Y-like; circRNAs, circular RNAs; CLASP1, Cytoplasmic linker associated protein 1; EGFR, Epidermal growth factor receptor; FAF1, Fas-Associated factor 1; FBXW7-185aa, A 185-amino acid protein encoded by circFBXW7; FBXW7, F-Box/WD repeat-containing protein 7; HIPK3, Homeodomain interacting protein kinase 3; NF1, Neurofibromin 1; NR, Not reported; PSMA1, Proteasome subunit alpha type 1; PRMT5, Protein arginine methyltransferase 5; RHOT1, Ras homolog family member T1; RPAP2, RNA polymerase II associated protein 2; STIL, SCL-interrupting locus protein; STAT3, Signal transducer and activator of transcription 3; TNBC, Triple-negative breast cancer; AUC values and sensitivity/specificity were derived from ROC analyses as reported in the original studies. Sample Size indicates the number of patients/controls in each study. Validation: “Yes” indicates validation in an independent external cohort; “Internal” indicates validation within the same study; “No” indicates no validation was performed. Comparison with Conventional Biomarkers: “Superior to CEA/CA15-3” indicates superior diagnostic performance; “Not compared” indicates no direct comparison was performed. BC Subtype: “Not specified” indicates that subtype information was not available in the original study. For hsa_circ_0000091, AUC values are derived from (1) a three-circRNA panel (hsa_circ_0000091, hsa_circ_0067772, hsa_circ_0000512) and (2) combined with ultrasound for predicting axillary lymph node metastasis. For hsa_circ_0104824, tissue expression was reported as downregulated, whereas plasma levels were upregulated. The mechanisms listed for circEGFR correspond to different functional contexts: IGF2BP2/SOX2 in doxorubicin resistance, miR-1299/EGFR in pirarubicin resistance, and miR-224-5p/ATG13/ULK1/ANXA2/TFEB in autophagy regulation.

### 5.1 CircRNAs as Diagnosis and Prognosis Biomarkers for BC

Several circRNAs have demonstrated high diagnostic accuracy in BC, with circulating AUC values exceeding 0.85. An AUC above 0.90 is generally considered to indicate excellent diagnostic performance, while values between 0.80 and 0.90 reflect good clinical utility [98]. circRASSF2 was significantly upregulated in both BC tissues and serum, with a serum AUC of 0.932. Its elevated expression was associated with shorter overall survival (OS) and progression-free survival (PFS) [67]. circCLASP1 was also overexpressed in BC tissues and serum, yielding AUC values of 0.8196 and 0.8902, respectively, and its expression correlated with lymph node metastasis, Ki-67 (proliferation marker) index, and tumor size [68]. Conversely, circ-FAF1 was significantly downregulated in BC serum relative to controls; reduced circ-FAF1 levels were associated with lymph node spread, distant metastasis, estrogen receptor-negative status, and poor prognosis, yielding an AUC of 0.885 (sensitivity 81.67%, specificity 76.67%) [69].

Multiple circRNAs showed AUC values between 0.80 and 0.85. hsa_circ_0008673 displayed an AUC of 0.833, surpassing cancer antigen 15-3 (CA15-3) (0.697) and carcinoembryonic antigen (CEA) (0.520), and its combination with these conventional markers further improved the AUC to 0.896 [70]. circRHOT1 was markedly elevated in serum exosomes of BC patients, yielding an AUC of 0.830 [71]. Hsa_circ_0000091 (circRPAP2) [72] exhibited an AUC of 0.825 in plasma, and 0.808 when combined with ultrasound for predicting axillary lymph node metastasis; notably, its combination with two additional circRNAs (hsa_circ_0067772 and hsa_circ_0000512) formed a diagnostic panel with an AUC of 0.974, a sensitivity of 97.1%, and a specificity of 90.2% [73].

Additional circRNAs with AUC values below 0.80 have also been documented. Hsa_circ_0001785 was significantly elevated in BC plasma (AUC = 0.784), showing superior diagnostic performance to CEA and CA15-3 [77], and acted as a miRNA sponge through the miR-942/suppressor of cytokine signaling 3 (SOCS3) axis [78]. circCDYL was upregulated in BC serum (AUC = 0.789, sensitivity 71.1%, specificity 75.5%) and correlated with poor prognosis via the miR-1275/autophagy-Related gene 7 (ATG7)/Unc-51 like autophagy activating kinase 1 (ULK1) autophagy pathway [79]. By contrast, circFBXW7 (hsa_circ_0001451) was downregulated in serum and exerted tumor-suppressive functions in TNBC through miR-197-3p/F-Box/WD repeat-containing protein 7 (FBXW7) and FBXW7-185aa encoding [81]. circPSMA1 was upregulated in TNBC-derived exosomes and promoted tumor progression via the miR-637/RAC-alpha serine/threonine-protein kinase (Akt1)/β-catenin axis, with potential implications for prognosis and immune evasion [94]. Other candidate circRNAs with limited diagnostic data are listed in Table 2.

### 5.2 CircRNAs as Chemoresistance and Recurrence Biomarkers for BC

Several blood-based circRNAs have been implicated in chemotherapy resistance and disease recurrence in BC. circABCB10 was upregulated in BC serum and associated with paclitaxel resistance via the let-7a-5p/dual specificity phosphatase 7 (DUSP7) axis [81]. hsa_circ_0069094 (circ_0069094) was elevated in BC plasma and tissues [82], and has been linked to BC malignancy through multiple pathways, including miR-591/hexokinase 2 (HK2) [83], miR-661/high mobility group A1 (HMGA1) [84], and miR-758-3p/zinc finger protein 217 (ZNF217) [85], as well as to paclitaxel resistance via the miR-136-5p/tyrosine 3-monooxygenase/tryptophan 5-monooxygenase activation protein zeta (YWHAZ) axis [86]. Beyond paclitaxel-related resistance, other circRNAs have been involved in resistance to distinct chemotherapeutic agents. circEGFR was upregulated in TNBC plasma exosomes [89] and associated with doxorubicin resistance via the insulin-like growth factor 2 mRNA-binding protein 2 (IGF2BP2)/SRY-box transcription factor 2 (SOX2) axis [91]. circSTAT3 was elevated in TNBC tissues and plasma exosomes, and contributed to cisplatin resistance through the miR-671-5p/notch receptor 1 (NOTCH1) axis [95]. Besides, circSTIL was markedly upregulated in plasma exosomes from TNBC patients and mediated the inhibitory effects of pirarubicin (THP) on TNBC cell malignancy [96]. Furthermore, low serum levels of hsa_circ_0001361 (circ-0001361) were correlated with a favorable response to neoadjuvant chemotherapy; the hsa_circ_0001361/miR-491/fibroblast growth factor receptor 4 (FGFR4) axis was associated with ultrasound-assessed axillary response following neoadjuvant chemotherapy (NAC), suggesting a potential role in predicting treatment efficacy and recurrence [97].

### 5.3 Mechanistic Integration and Prioritization of circRNA Biomarkers

**Figure 3:**
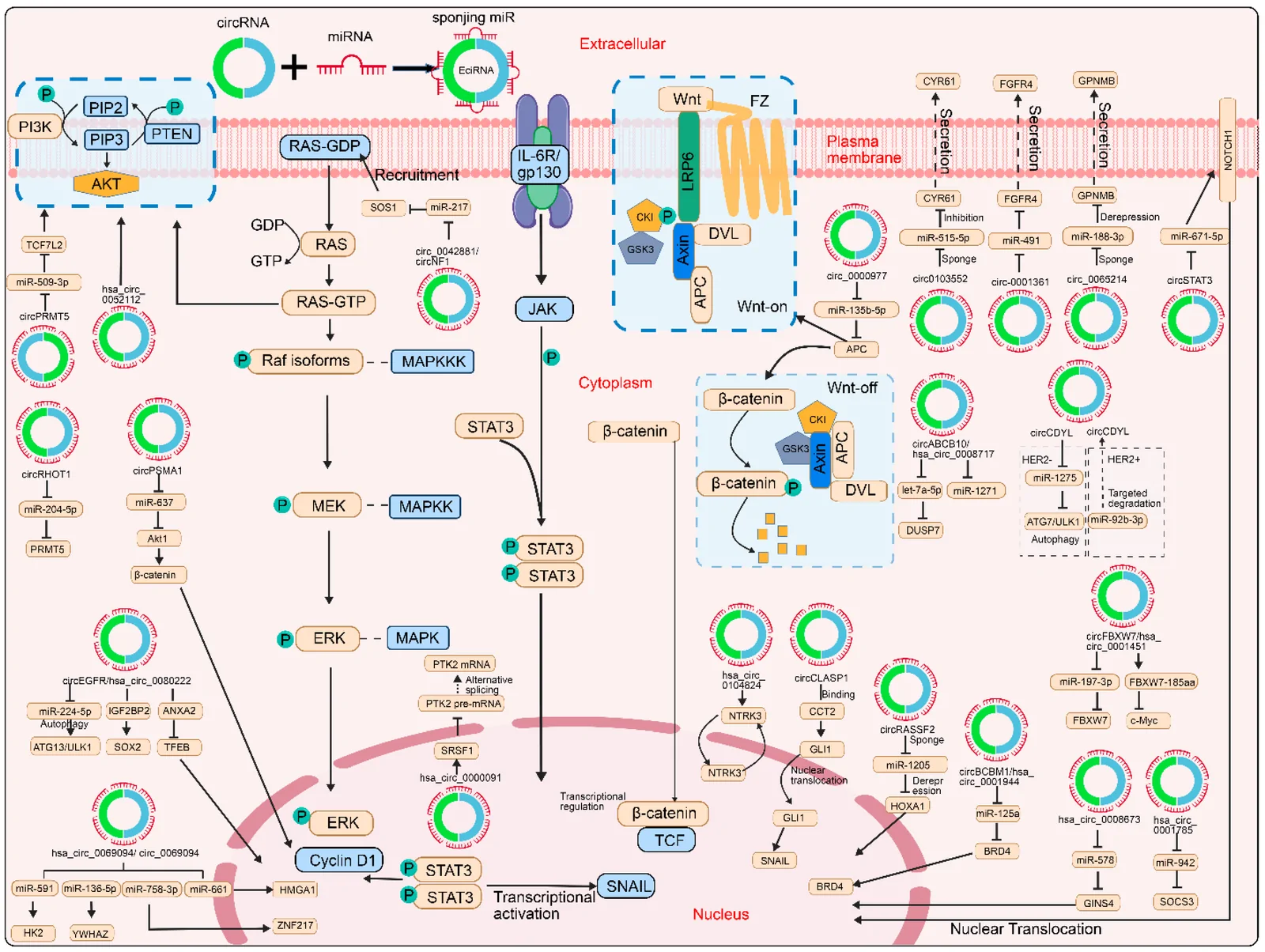
Integrated view of blood-based circular RNAs (circRNA)-mediated regulatory networks in breast cancer (BC) signaling pathways. Schematic illustration of key circRNAs derived from blood-based specimens (plasma, serum, or exosomes) and their convergence on major oncogenic signaling pathways in BC. CircRNAs are mapped onto their respective signaling nodes based on the evidence summarized in Table 2. Core pathways, including phosphoinositide 3-kinase (PI3K)/protein kinase B (AKT)/mechanistic target of rapamycin (mTOR), mitogen-activated protein kinase (MAPK)/extracellular signal-regulated kinase (ERK), and notch receptor 1 (NOTCH1) signaling, are highlighted as central hubs through which multiple circRNAs exert their regulatory effects. Representative circRNAs such as circPRMT5, circEGFR, circSTAT3, and circABCB10 are positioned at their corresponding pathway intersections. Arrows and T-bars denote activation and inhibition, respectively. This integrated view illustrates how diverse blood-based circRNA aberrations collectively contribute to systems-level reprogramming in BC. Abbreviations: ABCB10, ATP-binding cassette subfamily B member 10; AKT, Protein kinase B; Akt1, RAC-alpha serine/threonine-protein kinase; ANXA2, Annexin A2; APC, Adenomatous polyposis coli; ATG7, Autophagy-Related gene 7; ATG13, Autophagy-related protein 13; AUC, the area under the curve; BCBM1, Breast cancer brain metastasis 1; BC, Breast cancer; BRD4, Bromodomain-containing protein 4; CCT2, Chaperonin containing TCP1 subunit 2; CDYL, Chromodomain Y-like; circRNAs, circular RNAs; CKI, Casein kinase I; CLASP1, Cytoplasmic linker associated protein 1; c-myc, Cellular myelocytomatosis oncogene (or MYC proto-oncogene); CYR61, Cysteine-rich angiogenic inducer 61; DVL, Dishevelled segment polarity protein; DUSP7, Dual specificity phosphatase 7; EGFR, Epidermal growth factor receptor; ERK, Extracellular signal-regulated kinase; FAF1, Fas-Associated factor 1; FBXW7, F-Box/WD repeat-containing protein 7; FGFR4, Fibroblast growth factor receptor 4; FZ, frizzled; GDP, Guanosine diphosphate; GINS4, GINS complex subunit 4; GLI1, Glioma-Associated oncogene homolog 1; gp130, glycoprotein 130; GPNMB, Glycoprotein non-metastatic melanoma protein B; GSK3, Gycogen synthase kinase 3; GTP, Guanosine triphosphate; HER2, Human epidermal growth factor receptor 2; HIPK3, Homeodomain interacting protein kinase 3; HK2, Hexokinase 2; HMGA1, High mobility group A1; HOXA1, Homeobox A1; hsa, Homo sapiens; IGF2BP2, Insulin-like growth factor 2 mRNA-binding protein 2; IL-6R, Interleukin 6 receptor; LRP6, Low-density lipoprotein receptor-related protein 6; JAK, Janus kinase; MAPKKK, Mitogen-activated protein kinase kinase kinase; MAPKK, Mitogen-activated protein kinase kinase; MEK, Mitogen-activated protein kinase kinase; miRNA, microRNA; NF1, Neurofibromin 1; NTRK3, Neurotrophic receptor tyrosine kinase 3; NOTCH1, Notch receptor 1; NR, Not reported; PIP2, Phosphatidylinositol 4,5-bisphosphate; PIP3, Phosphatidylinositol (3,4,5)-trisphosphate; PI3K, Phosphoinositide 3-kinase; PTEN, Phosphatase and tensin homolog; PTK2, Protein tyrosine kinase 2; PSMA1, Proteasome subunit alpha type 1; PRMT5, Protein arginine methyltransferase 5; RAF, The rapidly accelerated fibrosarcoma; RAS, Rat sarcoma; RASSF2, Ras association domain-containing protein 2; RHOT1, Ras homolog family member T1; RPAP2, RNA polymerase II associated protein 2; STIL, SCL-interrupting locus protein; SOS1, Son of sevenless 1; SOX2, SRY-box transcription factor 2; SOCS3, Suppressor of cytokine signaling 3; SNAIL, Snail family transcriptional repressor 1; SRSF1, Serine and arginine rich splicing factor 1; STAT3, Signal transducer and activator of transcription 3; TCF7L2, Transcription factor 7 like 2; TFEB, Transcription factor EB; ULK1, Unc-51 like autophagy activating kinase 1; Wnt, Wingless-related integration site; YWHAZ, tyrosine 3-monooxygenase/tryptophan 5-monooxygenase activation protein zeta; ZNF217, Zinc finger protein 217. Image created with BioGDP.com.

BC progression is governed by the dysregulation of several core signaling pathways that control cell proliferation, survival, metastasis, and therapy resistance [99]. The pathways most frequently modulated by the circRNAs summarized in Table 2 include the phosphoinositide 3-kinase (PI3K)/protein kinase B (AKT)/mechanistic target of rapamycin (mTOR) and rat sarcoma (RAS)/mitogen-activated protein kinase (MAPK)/extracellular signal-regulated kinase (ERK) cascades, with emerging evidence also linking certain circRNAs to NOTCH1 signaling [100]. For instance, circPRMT5 (upregulated in serum) activates PI3K/AKT signaling via the miR-509-3p/transcription factor 7 like 2 (TCF7L2) axis [88], whereas circEGFR (upregulated in TNBC plasma exosomes) has been implicated in THP resistance through the miR-1299/EGFR axis [90]. circSTAT3 contributes to cisplatin resistance via the miR-671-5p/NOTCH1 axis [95], and circABCB10 (upregulated in serum) is implicated in paclitaxel resistance through the let-7a-5p/DUSP7 axis [81]. Recognition of these shared pathways provides a mechanistic framework for understanding circRNA function and highlights potential targets for therapeutic intervention. An integrated view of these circRNA-mediated regulatory networks is presented in Fig. 3.

While the summarized data provide a comprehensive list of candidate biomarkers, a subset of circRNAs merit particular attention based on their diagnostic performance and functional relevance. In particular, hsa_circ_0008673 [70] and circRASSF2 [67] stand out, as their diagnostic accuracy and characterized mechanistic roles support further clinical evaluation. Conversely, other candidates with high diagnostic accuracy, such as circCLASP1 [68] and circ-FAF1 [69], require independent validation in larger, multicenter cohorts before their clinical utility can be confirmed. This prioritization may help direct future investigations toward the most translationally promising circRNA biomarkers.

Several circRNAs merit particular attention based on their diagnostic performance and mechanistic relevance. Among them, hsa_circ_0008673 and hsa_circ_0001785 have demonstrated high diagnostic accuracy with well-characterized mechanistic roles. Their diagnostic performance has been evaluated in head-to-head comparisons with conventional serum biomarkers. For instance, hsa_circ_0008673 achieved an AUC of 0.833 in a cohort of 378 BC patients and 102 healthy controls [70], while hsa_circ_0001785 outperformed CEA (0.562) and CA15-3 (0.639) with an AUC of 0.784 in the same cohort [77]. However, most circRNA studies lack comparisons with cancer antigen 27.29 (CA 27.29), and the available evidence remains exploratory. Nonetheless, the superiority of certain circRNAs over CEA and CA15-3 suggests their potential as complementary biomarkers. Other candidates with high diagnostic accuracy, such as circCLASP1 and circ-FAF1, require independent validation in larger cohorts before clinical application.

A critical evaluation of the literature reveals marked heterogeneity in the reported expression levels and diagnostic performance of specific circRNAs. For instance, although hsa_circ_0001785 has been proposed as a diagnostic biomarker in certain studies, its functional role appears contradictory, with evidence indicating both tumor-suppressive and oncogenic properties depending on the cellular context. Such inconsistencies may arise from several factors, including the use of different sample types such as plasma versus serum, variable storage conditions, and divergent RNA extraction protocols, normalization methods, and detection platforms ranging from RT-qPCR to RNA-seq [101]. Differences in patient cohort characteristics, including tumor stage, molecular subtype, and prior treatment history, may further contribute to the variability in circRNA expression profiles. The absence of standardized experimental protocols and universally accepted cut-off values represents a major obstacle to assessing the reproducibility of circRNA biomarker studies [102].

A direct comparison of AUC values across studies may be biased, as each study employed distinct patient cohorts, inclusion criteria, and statistical models. Differences in sample size, disease stage distribution, and control group composition can markedly affect AUC estimates. Accordingly, the AUC values presented in this review should be interpreted as study-specific performance metrics rather than directly comparable measures [103]. Future studies adopting standardized protocols and independent validation cohorts are needed to establish robust cross-study comparisons of these biomarkers.

## 6 CircRNAs Associated with Molecular Subtypes in BC Tissues

While tissue-based studies have uncovered subtype-specific circRNA signatures, whether these findings can be translated into non-invasive plasma-based subtyping tools remains unclear. In this section, we therefore summarize the current evidence on subtype-associated circRNA expression in BC tissues, which may help clarify the biological underpinnings of plasma circRNA signatures discussed in preceding sections. The following subsections describe circRNAs linked to the major molecular subtypes of BC, namely luminal A/B, human epidermal growth factor receptor 2 (HER2)-positive, and TNBC [104]. Luminal tumors are uniformly defined by estrogen receptor (ER) positivity in all circRNA studies reported to date, and accordingly, this review emphasizes circRNAs that are aberrantly expressed and functionally relevant in ER-positive BC. CircRNA expression profiles vary considerably across subtypes, with the majority of evidence derived from tissue-based analyses. Therefore, these subtype-restricted circRNA signatures not only reflect the molecular heterogeneity of BC but also provide a foundation for exploring subtype-specific biological pathways. A summary of representative circRNAs aberrantly expressed in each molecular subtype is presented in Table 3.

**Table 3:** Aberrantly expressed Circular RNAs (circRNAs) in molecular subtypes of breast cancer (BC) tissues.

CircRNAs	Subtype	Expression	Role	Category	Pathways	References
circFOXK2/hsa_circ_0000816	ER-positive	Upregulated	Oncogenic	tamoxifen resistance	CCND1/CDK4/6/p-RB-E2F; IGF2BP3/miR-370	[105,106]
circTNK2/hsa_circ_000937	ER-positive	Upregulated	Oncogenic	tamoxifen resistance	C-TNK2-487aa/STAT3/CXCL10	[107]
circPGR	ER-positive	Upregulated	Oncogenic	diagnostic biomarker	miR-301a-5p	[108]
circPVT1	ER-positive	Upregulated	Oncogenic	diagnostic biomarker; tamoxifen resistance	miR-181a-2-3p/ERα	[109]
hsa_circ_0087378	ER-positive	Downregulated	Tumor suppressive	diagnostic biomarker	miR-1260b/SFRP1	[110]
circEPSTI1/hsa_circ_0000479	HER2-positive; TNBC	Upregulated	Oncogenic	oncogenic factor; poor prognosis	miR-145/ERBB3; miR-4753, miR-6809/BCL11A	[111,112]
circ-ERBB2/hsa_circ_0007766	HER2-positive; TNBC	Upregulated	Oncogenic	oncogenic factor; auxiliary biomarker	miR-136-5p, miR-198/TFAP2C; miR-136-5p/PDK4	[113,114]
circCDYL2	HER2-positive	Upregulated	Oncogenic	Trastuzumab resistance	GRB7-FA K/AKT/ERK1/2	[115]
circWWC3	TNBC	Upregulated	Oncogenic	predictneoadjuvant response	CSF2	[116]
circSCAP	TNBC	Upregulated	Oncogenic	platinum resistance	PI3K/AKT	[117]
circPLK1	TNBC	Upregulated	Oncogenic	doxorubicin resistance	miR-940/ETS1	[118]
circPARPBP/hsa_circ_0000432	TNBC	Upregulated	Oncogenic	chemoresistance	SRCAP/CCL20	[119]
circ-BISC	TNBC	Downregulated	Tumor suppressive	BETi resistance reversal agent	c-MYC	[120]
circ_0001522/circ_0001278/circ_0001801	TNBC	Upregulated	Oncogenic	recurrence	miR-4458, miR-145-5p, miR-760/*CCND1*, *ROBO4*, *MMP1*	[121]
circFGFR4	TNBC	Upregulated	Oncogenic	anti-PD-1 immunotherapy resistance	miR-185-5p/CXCR4	[122]

Note: AKT, Protein kinase B; BC, Breast cancer; BCL11A, B-cell lymphoma/leukemia 11A; BISC, BETi sensitizing circRNA; CCND1, Cyclin D1; CCL20, Chemokine (C-C motif) ligand 20; CDK4, Cyclin-dependent kinase 4; CDYL2, Chromodomain Y like 2; CircRNAs, Circular RNAs; c-MYC, Cellular myelocytomatosis oncogene (or MYC proto-oncogene); CSF2, Colony stimulating factor 2; C-TNK2-487aa, C-terminal TNK2-encoded 487-amino acid protein; CXCL10, C-X-C motif chemokine ligand 10; CXCR4, C-X-C chemokine receptor Type 4; DOCK1, Dedicator of cytokinesis 1; EPSTI1, Epithelial stromal interaction 1; ETS1, E26 transformation-specific sequence 1; ERBB2, Erb-B2 receptor tyrosine kinase 2; ERBB3, Erb-B2 receptor tyrosine kinase 3; ER, Estrogen receptor; ERK, Extracellular signal-regulated kinase; FOXK2, Forkhead box protein K2; FGFR4, Fibroblast growth factor receptor 4; GRB7, Growth factor receptor bound protein 7; HER2, Human epidermal growth factor receptor 2; IGF2BP3, Insulin-like growth factor 2 mRNA-binding protein 3; MMP1, Matrix metalloproteinase 1; PARPBP, PARP1 binding protein; PD-1, Programmed cell death protein 1; PDK4, Pyruvate dehydrogenase kinase 4; PGR, Progesterone receptor; PI3K, Phosphoinositide 3-kinase; PLK1, Polo-like kinase 1; PVT1, Plasmacytoma variant translocation 1; p-RB-E2F, Phosphorylated retinoblastoma protein-E2F transcription factor; ROBO4, Roundabout guidance receptor 4; SCAP, SREBP cleavage-activating protein; SFRP1, Secreted frizzled-related protein 1; SRCAP, Snf2-related CREBBP activator protein; STAT3, Signal transducer and activator of transcription 3; TFAP2C, Transcription factor AP-2 gamma; TNBC, Triple-negative breast cancer; TNK2, Tyrosine kinase non receptor 2; WWC3, WW and C2 domain containing 3.

### 6.1 CircRNAs in ER-Positive BC

ER-positive BC, which encompasses the Luminal A and Luminal B molecular subtypes, accounts for approximately 70% of all BC cases. In the circRNA literature, the large majority of studies have classified patients based on ER status rather than gene expression-based subtyping to distinguish Luminal A from Luminal B. Accordingly, in this section, we adopt the terminology used in the original reports and refer to these cases as ER-positive BC, while acknowledging that they predominantly represent the Luminal subtypes.

Several circRNAs have been implicated in ER-positive BC progression and endocrine therapy resistance. circFOXK2 (hsa_circ_0000816) was identified as the most highly expressed circRNA in ER-positive tumor tissues and promoted tamoxifen resistance through stabilization of cyclin D1 (CCND1) mRNA and subsequent activation of the CCND1-Cyclin-dependent kinase 4/6 (CDK4/6)-phosphorylated retinoblastoma protein-E2F transcription factor (p-RB-E2F) axis; notably, Anti-sense oligonucleotide (ASO)-circFOXK2 restored tamoxifen sensitivity in resistant cells [105]. circTNK2 (hsa_circ_000937) was markedly overexpressed in tamoxifen-resistant tissues and correlated with poor prognosis; mechanistically, it exerted dual functions by encoding the C-tyrosine kinase non receptor 2 (TNK2)-487aa peptide to suppress the signal transducer and activator of transcription 3 (STAT3)/C-X-C motif chemokine ligand 10 (CXCL10) axis and mediate Natural Killer (NK) cell immune evasion, while also binding serine and arginine rich splicing factor 1 (SRSF1) to activate AKT-mTOR signaling and drive tamoxifen resistance [107]. circPGR, an estrogen-induced circRNA derived from the progesterone receptor (PGR) gene, was specifically upregulated in ER-positive BC and functioned as a miR-301a-5p sponge to promote cell cycle progression [108]. Similarly, circPVT1 was highly expressed in ER-positive tumors and upregulated ERα expression by sponging miR-181a-2-3p, thereby promoting tamoxifen resistance; it also bound mitochondrial antiviral signaling (MAVS) protein to suppress type I interferon signaling and facilitate immune evasion [109].

In contrast, hsa_circ_0087378 was markedly downregulated in ER-positive BC tissues and functioned as a tumor suppressor through the miR-1260b-SFRP1 axis, inhibiting estrogen pathway activity and cell proliferation [110]. Notably, the functional role of this circRNA appears to be cell-type-dependent, as it has also been reported to act as an oncogene in esophageal squamous cell carcinoma [123] and non-small cell lung cancer [124]. Collectively, these findings suggest that circRNAs in ER-positive BC encompass both oncogenic and tumor-suppressive roles, with several candidates directly linked to endocrine therapy response and immune modulation.

### 6.2 CircRNAs in HER2-Positive BC

Accumulating evidence has linked circRNAs to HER2-positive BC progression and trastuzumab resistance. circEPSTI1 (hsa_circ_0000479) was significantly overexpressed in HER2-positive tissues and cell lines, where it promoted proliferation and invasion through the miR-145/ERBB3 axis [111]. circ-ERBB2 (hsa_circ_0007766), derived from back-splicing of the erb-B2 receptor tyrosine kinase 2 (ERBB2) gene, was upregulated in HER2-positive tumors and facilitated proliferation and metastasis by sponging miR-136-5p and miR-198, leading to transcription factor AP-2 gamma (TFAP2C) activation and downstream PI3K/AKT and MAPK signaling; knockdown of circ-ERBB2 substantially suppressed tumor growth *in vivo* [113].

In addition to their roles in proliferation and metastasis, certain circRNAs directly contribute to trastuzumab resistance. circCDYL2 was significantly upregulated in trastuzumab-resistant HER2-positive BC and correlated with larger tumor dimensions, lymph node metastasis, and reduced disease-free survival (DFS) and OS in a cohort of 127 patients. Mechanistically, circCDYL2 stabilized growth Factor Receptor Bound Protein 7 (GRB7) by suppressing its ubiquitination-mediated degradation, thereby enhancing GRB7-focal adhesion kinase (FA K) binding and sustaining AKT and ERK1/2 activation, which allowed HER2-positive cells to evade trastuzumab inhibition. Notably, treatment with FA K or GRB7 inhibitors restored drug sensitivity in resistant cells, suggesting the therapeutic potential of targeting circCDYL2 or the GRB7/FA K axis [115]. Collectively, these findings indicate that circRNAs in HER2-positive BC act as oncogenic drivers and represent promising therapeutic targets, particularly for overcoming trastuzumab resistance.

### 6.3 CircRNAs in TNBC

TNBC is known for its aggressive clinical course and poor prognosis, with multiple circRNAs involved in tumor progression and therapy resistance. circEPSTI1 was overexpressed in TNBC tissues and linked to larger tumor size, lymph node metastasis, and shorter OS through miR-4753 and miR-6809 sponging and subsequent B-cell lymphoma/leukemia 11A (BCL11A) upregulation [112]. circ-ERBB2 facilitated proliferation and glycolysis in TNBC via the miR-136-5p/pyruvate dehydrogenase kinase 4 (PDK4) axis, promoting a metabolic shift toward aerobic glycolysis [114]. circWWC3 was upregulated in TNBC and interacted with Vimentin to regulate colony stimulating factor 2 (CSF2) secretion; its expression was also integrated into a predictive nomogram for pathological complete response (pCR) after neoadjuvant therapy [116]. A cluster of three circRNAs, including circ_0001522, circ_0001278, and circ_0001801, was associated with increased recurrence risk in a cohort of 96 TNBC patients through sponging miR-4458, miR-145-5p, and miR-760 to upregulate CCND1, roundabout guidance receptor 4 (ROBO4), and matrix metalloproteinase 1 (MMP1); knockdown of these circRNAs reduced colony formation by up to 70% in TNBC cells [121].

Beyond their roles in tumor progression, multiple circRNAs are critically involved in therapeutic resistance in TNBC. circSCAP was elevated in platinum-resistant TNBC and encoded the SREBP cleavage-activating protein (SCAP)-129aa peptide, which stabilized phosphoinositide-3-kinase regulatory subunit 2 (PIK3R2) and activated PI3K/AKT signaling, thereby driving platinum resistance [117]. circPLK1 conferred resistance to the anthracycline doxorubicin by sponging miR-940 to upregulate E26 transformation-specific sequence 1 (ETS1); its high expression, particularly when combined with elevated ETS1, was associated with the lowest pCR rates in a cohort of 240 TNBC patients [118]. circPARPBP interacted with the snf2-related CREBBP activator protein (SRCAP) complex to activate chemokine (C-C motif) ligand 20 (CCL20) transcription, promoting stemness and chemoresistance; the natural compound isoliquiritigenin was reported to inhibit this axis and overcome chemoresistance in preclinical models [119]. In the context of targeted therapy, circ-BISC functioned as a tumor suppressor by targeting insulin-like growth factor 2 mRNA-binding protein 2 (IGF2BP2) to overcome acquired bromodomain and extra-terminal domain inhibitor (BETi) resistance, and its combination with the BETi OTX-015 displayed enhanced efficacy in resistant models [120]. circFGFR4 contributed to immune evasion and anti-programmed cell death protein 1 (PD-1) resistance through the miR-185-5p/C-X-C Chemokine Receptor Type 4 (CXCR4) axis, which reduced CD8^+^ T cell infiltration and established an immunosuppressive microenvironment [122].

Taken together, these findings underscore the diverse functions of circRNAs in TNBC, with a substantial subset directly involved in resistance to chemotherapy, targeted therapy, and immunotherapy. Notably, many of these circRNAs converge on shared signaling hubs, including PI3K/AKT, glycolysis, and immune checkpoint pathways, suggesting that they operate as part of broader regulatory networks rather than in isolation. Considering the intrinsic heterogeneity of TNBC, circRNA-based classifiers that distinguish distinct resistance mechanisms may enhance patient stratification and inform personalized therapeutic approaches. Future research should focus on validating these circRNA signatures in large, prospective cohorts and assessing their additive value alongside established clinicopathological parameters to improve clinical decision-making in TNBC management.

## 7 CircRNA-Targeted Therapeutic Strategies in BC: Current Approaches and Preclinical Evidence

Beyond their biomarker potential, circRNAs are increasingly regarded as directly actionable targets in BC. The circRNAs discussed above display subtype-restricted expression patterns and are functionally associated with tumor progression and drug resistance, supporting the rationale for their therapeutic manipulation. Current approaches fall broadly into two categories: loss-of-function and gain-of-function strategies [125]. Together, these strategies have shown preclinical efficacy in ER-positive, HER2-positive, and TNBC models, laying the groundwork for future clinical translation.

### 7.1 Loss-of-Function Strategies

Loss-of-function strategies are designed to counteract oncogenic circRNAs that are highly expressed in BC. Antisense oligonucleotides (ASOs) represent one such approach. These short synthetic nucleic acids bind complementary RNA sequences with high specificity, and upon binding, they can block functional sites on circRNAs, thereby disrupting circRNA-protein or circRNA-RNA interactions and suppressing downstream biological activities [126]. A major advantage of ASOs lies in their ability to recognize the back-splice junction (BSJ), a sequence element present exclusively in circRNAs and absent from their linear counterparts, which enables selective circRNA knockdown without affecting host gene expression [127]. In ER-positive BC models, ASO-circFOXK2 administration inhibited tumor cell proliferation *in vitro* and *in vivo* and restored tamoxifen responsiveness in resistant cells [105]. Similarly, ASO-mediated circPVT1 silencing suppressed ER-positive BC cell growth and tumor formation while overcoming tamoxifen resistance [109]. The applicability of this strategy is further illustrated by circCDYL2 in HER2-positive BC, where ASO-mediated silencing disrupted the circCDYL2-GRB7-FA K complex and overcame trastuzumab resistance [115].

RNA interference (RNAi) offers another route for circRNA suppression via small interfering RNAs (siRNAs) and short hairpin RNAs (shRNAs). After entering cells, siRNAs are incorporated into the RNA-induced silencing complex (RISC), which recognizes and cleaves the target circRNA. shRNAs designed against back-splice junctions have also been used for circRNA knockdown; however, inconsistencies between shRNA-mediated silencing and phenotypic effects have been observed, raising questions about their specificity and reliability [128]. The utility of RNAi-based circRNA silencing has been well documented in BC. For example, siRNA-mediated knockdown of circ_0001522, circ_0001278, and circ_0001801 significantly suppressed proliferation, colony formation, and migration in TNBC cells [121]. Likewise, shRNA-mediated knockdown of circESR1 inhibited cell cycle progression in ER-positive BC cells [129]. Additionally, shRNA-mediated circ-ERBB2 silencing substantially reduced tumor growth in HER2-positiveBC xenografts [113]. These examples underscore the broad applicability of RNAi strategies for functional studies and therapeutic targeting of circRNAs in BC.

DNAzymes offer a catalytic approach for RNA cleavage. These single-stranded DNA molecules recognize and cleave specific RNA sequences via a metal-ion-dependent mechanism. Their intrinsic catalytic activity and programmability have been harnessed for both RNA knockdown and biosensing, including recent applications in the detection of cancer-associated circRNAs [130]. In BC research, DNAzymes have primarily been used as highly sensitive tools for circRNA detection. For example, a ligation-controlled single-molecule biosensor based on Exponential Amplification Reaction (EXPAR)-induced DNAzyme generation has been developed for attomolar-level detection of circFOXO3, enabling discrimination between BC tissues and normal counterparts [131]. Although DNAzyme-mediated circRNA suppression has been validated in other cancer types, its application as a loss-of-function tool for functional studies of specific circRNAs in BC remains largely unexplored.

The clustered regularly interspaced short palindromic repeats (CRISPR)-CRISPR-associated protein 13d (Cas13d) system has recently emerged as a powerful tool for specific circRNA degradation. Unlike CRISPR-associated protein 9 (CRISPR-Cas9), which targets DNA, Cas13 is an RNA-targeting nuclease that, when programmed with a BSJ-specific guide RNA, can specifically bind and cleave target circRNAs without affecting host gene expression. Optimized CRISPR-Cas13d technology has demonstrated substantially enhanced specificity of circRNA silencing compared with traditional shRNA methods [128]. In BC, the utility of CRISPR-Cas13d has been validated in both functional screening and therapeutic targeting. For example, a large-scale CRISPR-Cas13d screen identified circFAM120A as an oncogenic circRNA that promotes TNBC cell proliferation by sequestering IGF2BP2, thereby upregulating FAM120A expression [132]. Similarly, CRISPR-Cas13d-mediated silencing of circ-β-TrCP effectively suppressed cell viability in trastuzumab-resistant HER2-positive BC cells, highlighting the feasibility of this approach for overcoming drug resistance [133]. Overall, these findings support the potential of CRISPR-Cas13d for both functional genomics and therapeutic applications in BC.

### 7.2 Gain-of-Function Strategies

Gain-of-function strategies aim to restore tumor-suppressive circRNAs that are downregulated in BC. This can be achieved by delivering synthetic circRNAs or circRNA-overexpressing plasmids into target cells. A major hurdle lies in achieving efficient and targeted delivery to tumor tissues. Currently, two principal approaches are employed for circRNA delivery *in vivo*: non-viral vectors, including lipid nanoparticles (LNPs) and engineered exosomes, and viral vectors [134]. LNPs are among the most advanced nucleic acid delivery platforms, capable of encapsulating and delivering circRNAs efficiently, with demonstrated potential in BC models. Exosomes, as naturally secreted nanoscale vesicles, represent attractive delivery vehicles owing to their biocompatibility and efficient cellular uptake. Lentivirus-based systems allow the introduction of circRNA-overexpressing plasmids into cells, enabling sustained and stable expression [135].

The utility of LNPs for circRNA delivery has been demonstrated in preclinical BC models. One example is LNP-encapsulated circular adenosine deaminase acting on RNA (ADAR)-recruiting RNAs (Circ-arRNA), which were designed to correct TP53 nonsense mutations in TNBC 4T1 cells and xenograft models, achieving mutation correction rates of 73.32% *in vitro* and 48.48% *in vivo* [136]. Another case involves immunocyte-tropic LNPs delivering circRNACAR, which encodes chimeric antigen receptor (CAR) proteins and enables *in vivo* generation of panCAR cells, including CAR-T cells, CAR-natural killer (CAR-NK) cells, and CAR-macrophages [137]. This strategy substantially suppressed tumor growth and remodeled the tumor microenvironment in mouse models, including 4T1-HER2 BC models. Furthermore, combining *in vivo* pan-chimeric antigen receptor (panCAR) with circRNA-based vaccines encoding HER2 antigens yielded synergistic antitumor effects. Together, these observations underscore the broad applicability and translational promise of LNP-mediated circRNA delivery in BC therapy.

Exosomes are naturally secreted nanoscale vesicles that have emerged as attractive delivery vehicles owing to their stability, low immunogenicity, and capacity for targeted cellular uptake. Engineered exosomes preloaded with therapeutic circRNAs offer a novel direction for personalized BC medicine. For instance, exosomal delivery of circRNA-CREIT enhanced doxorubicin sensitivity in TNBC cells through PKR destabilization and suppression of stress granule assembly. *In vivo*, this approach reduced tumor growth and increased apoptosis in xenograft models [138]. In another study, circSTIL was found to mediate the antitumor effects of pirarubicin in TNBC cells, and its expression in plasma exosomes holds diagnostic and prognostic value [96]. Collectively, exosomal circRNAs in BC are clinically relevant as circulating biomarkers for diagnosis, prognosis, and treatment monitoring, as mediators of tumor progression and drug resistance, and as potential nanocarriers for targeted therapies.

Lentiviral vectors enable efficient genomic integration of circRNA overexpression constructs, supporting sustained gene regulation and functional interrogation of circRNAs in BC initiation, progression, and drug resistance. For example, lentivirus-mediated circHSDL2 overexpression promoted BC cell proliferation, migration, and invasion via the miR-7978/zinc finger protein 704 (ZNF704)/Hippo axis, as validated in cellular and animal models [139]. Moreover, lentivirus-mediated circ_0008536 overexpression resensitized doxorubicin-resistant TNBC cells to chemotherapy via the miR-382-5p/gametogenetin binding protein 2 (GGNBP2) axis, with concomitant suppression of tumor growth in xenograft models [140]. These examples underscore the utility of lentiviral systems for gain-of-function studies in BC circRNA biology.

## 8 Discussion

### 8.1 Potential of CircRNAs as Companion Biomarkers for Therapy Selection and Subtype Stratification

The treatment landscape for TNBC has been transformed by the KEYNOTE-522 trial, which established neoadjuvant chemoimmunotherapy as the standard of care for high-risk early-stage disease [141]. This paradigm shift underscores the pressing need for predictive biomarkers to guide patient selection and treatment optimization. Growing evidence supports the utility of specific circRNAs as companion biomarkers for therapy selection in BC. In ER-positive disease, circFOXK2 and circPVT1 were upregulated in tamoxifen-resistant cells and correlated with poor endocrine response [105,109]. In HER2-positive disease, circCDYL2 was implicated in trastuzumab resistance, suggesting its value in identifying patients unlikely to benefit from anti-HER2 therapy [115]. In TNBC, downregulation of circ-BISC was associated with BET inhibitor resistance, and its restoration overcame resistance, supporting its predictive potential for BET inhibitor response [120]. Similarly, circFGFR4 expression correlated with resistance to anti-PD-1 immunotherapy, pointing to its possible role in guiding treatment decisions [122]. In the context of neoadjuvant chemotherapy, circWWC3 expression was incorporated into a nomogram for predicting pCR in TNBC [116].

For subtype stratification, circRNAs show expression patterns that are largely specific to particular molecular subtypes. circFOXK2 and circPGR were preferentially expressed in ER-positive BC, whereas hsa_circ_0087378 was downregulated in this subtype. circCDYL2 was enriched in HER2-positive tumors. A broader array of dysregulated circRNAs has been reported in TNBC, including circWWC3, circSCAP, and circPLK1. This subtype-restricted expression profile indicates that circRNA-based classifiers may assist in molecular subtyping, particularly when conventional immunohistochemistry is inconclusive. However, the majority of ER-positive studies have not distinguished between Luminal A and Luminal B subtypes, largely because they relied on immunohistochemical rather than gene expression-based classification. As a result, whether circRNA expression profiles differ between Luminal A and Luminal B tumors remains unknown, underscoring an unmet need that merits further exploration.

Although certain plasma circRNAs have displayed superior diagnostic performance compared with CEA and CA15-3 in BC, they should be considered complementary rather than competitive to these conventional markers [142]. This integration with existing biomarkers holds promise for improving diagnostic accuracy, as reflected by the increased AUC of 0.839 observed when hsa_circ_0001785 was combined with CEA and CA15-3 [77]. Overall, the circRNAs discussed in this review constitute a flexible biomarker platform with potential utility across multiple clinical scenarios in BC, including subtype classification, treatment selection, and longitudinal monitoring of disease progression and therapeutic response. The eventual adoption of circRNA-based testing into routine practice could support more precise and personalized care for BC patients.

Beyond their utility as individual biomarkers, circRNAs should be evaluated in the broader context of liquid biopsy analytes [143]. Circulating tumor DNA (ctDNA) is the most clinically established liquid biopsy approach for BC, with proven value in risk stratification, treatment monitoring, and resistance mutation detection [144]. Nevertheless, ctDNA and circRNAs provide distinct biological information: ctDNA reflects genomic alterations and tumor heterogeneity, whereas circRNAs capture dynamic changes in gene expression and regulatory networks [145]. A recent large-scale pan-cancer study reported that adding ctRNA, which includes circRNAs, to ctDNA-based liquid biopsy increased the actionable diagnostic yield by 36.7% [146]. This supports integrating circRNAs into multi-analyte panels alongside ctDNA, as they may uncover resistance mechanisms not readily detectable by DNA-based approaches.

### 8.2 Complementary Role of Imaging and CircRNA Biomarkers

Mammography remains the standard screening tool for BC; however, its sensitivity is considerably diminished in women with dense breasts, where fibroglandular tissue may obscure lesions and generate false-negative findings [147]. Breast ultrasound (US) serves as a useful adjunct, particularly for women with dense breasts or those at higher risk, as it avoids ionizing radiation and is more suitable for repeated examinations. While US improves cancer detection in dense breasts, it is accompanied by an increased false-positive rate [148]. Automated breast US (ABUS) and handheld US (HHUS) have been reported to identify approximately 2 to 3 additional malignancies per 1000 screenings in women with dense breasts [149]. Magnetic resonance imaging (MRI) offers the highest sensitivity among available imaging modalities for BC detection and is recommended for high-risk populations, including BRCA1/2 mutation carriers and women with extremely dense breasts. Supplemental MRI has been shown to detect about 19 additional cancers per 1000 screenings in women with dense breasts, markedly outperforming ultrasound or digital breast tomosynthesis [150]. Despite its superior sensitivity, MRI has several drawbacks, such as higher false-positive rates, prolonged acquisition times, dependence on contrast agents, substantial costs, and restricted accessibility.

Despite these advances, the direct integration of circRNA biomarkers with imaging modalities in BC remains relatively underexplored, although supportive evidence is emerging for their complementary utility. For example, the circRNA-0001361/miR-491/FGFR4 axis has been correlated with ultrasound-assessed axillary response following neoadjuvant chemotherapy [97], and the circ_PRDM5/miR-25-3p/ankyrin repeat domain 46 (ANKRD46) axis has been associated with ultrasound-assessed malignant behaviors [151]. Additionally, plasma levels of hsa_circ_0000091 were correlated with axillary lymph node metastasis and prognosis, and its combination with ultrasound yielded an AUC of 0.808 for predicting axillary lymph node metastasis [73]. Collectively, these observations suggest that circRNA expression patterns may reflect imaging-detectable tumor characteristics, reinforcing the rationale for integrating molecular and imaging biomarkers to improve BC diagnosis and prognostication.

Imaging modalities and circulating circRNA biomarkers serve as complementary non-invasive tools for BC assessment. Specifically, imaging supplies structural and phenotypic data regarding tumor presence and characteristics, whereas circRNAs deliver molecular information relevant to subtype classification and therapeutic response prediction. Combining imaging features with circRNA signatures could facilitate earlier detection and improve risk stratification, particularly in high-risk patients for whom imaging alone is inadequate. This synergy supports the creation of integrated diagnostic approaches that merge anatomical and molecular data, thereby strengthening clinical decision-making in BC care.

## 9 Limitations, Challenges, and Future Directions of circRNA Biomarkers

Despite the encouraging preclinical evidence summarized above, the clinical translation of circRNA-based biomarkers in BC faces considerable hurdles, including a shortage of standardized assays and reference materials, alongside the pressing need for large-scale prospective validation studies. Beyond these technical and validation challenges, three additional issues warrant particular consideration: the development of subtype-specific plasma circRNA signatures, the incorporation of circRNAs into multi-omic liquid biopsy panels, and the persistent challenge of disease specificity [152,153].

### 9.1 Methodological and Clinical Limitations

Evaluating current evidence on circRNA biomarkers in BC reveals several limitations. Many studies have small sample sizes and lack independent validation, increasing the risk of overfitting and limiting generalizability. Methodological heterogeneity, encompassing variations in sample processing, detection platforms, and normalization strategies, may contribute to inconsistent findings. This issue is compounded by the diversity of biospecimen types used, such as plasma, serum, whole blood, and exosomes, yet the comparability of circRNA measurements across these materials remains poorly defined due to the absence of standardized protocols. Furthermore, a persistent disconnect exists between tissue-based discovery and plasma-based validation. Although subtype-specific circRNA expression is consistently documented in BC tissues, most plasma-based studies have not stratified patients by molecular subtype, and circulating profiles do not reliably mirror tissue expression patterns. Disease specificity also remains a major concern. For example, hsa_circ_0001944 is elevated in BC [51] as well as in leukemia [53], gastric cancer [52], and non-small cell lung cancer [154], limiting its utility as a BC-specific marker. These limitations collectively point to a central unresolved issue, namely the translation of tissue-based findings to plasma-based applications.

### 9.2 Challenges for Clinical Translation

The development of subtype-specific plasma circRNA signatures remains a key priority, as such non-invasive tools can overcome the limitations of tissue-based classification and enable dynamic monitoring of tumor evolution. Although numerous circRNAs are aberrantly expressed in BC tissues across molecular subtypes, their utility for non-invasive subtyping has yet to be systematically examined [155]. Several candidate circRNAs have shown initial promise in patient-derived plasma exosomes. For example, circEGFR is upregulated in TNBC tissues and has been detected at higher levels in plasma-derived exosomes [89]. Similarly, circSTAT3 is elevated in TNBC tissues and patient plasma exosomes [95]. These findings suggest that subtype-specific circRNA expression signatures identified in tissues may be detectable in circulation and offer a potential route toward non-invasive molecular subtyping. Nonetheless, large-scale profiling studies are needed to establish robust signatures that could refine current immunohistochemistry-based classification, especially when tissue biopsy is challenging or yields equivocal results.

A related yet more ambitious challenge is the integration of circRNAs into multi-omic liquid biopsy panels. The combination of circRNAs with ctDNA, circulating tumor cells (CTCs), and exosomal proteins could enhance diagnostic sensitivity and specificity, refine treatment monitoring, and facilitate earlier detection of resistance. However, substantial computational and biological challenges, including data normalization, batch correction, and the development of interpretable algorithms, must be addressed to realize this potential [156].

### 9.3 Future Directions for Clinical Translation

To overcome these challenges, future studies should prioritize independent validation in large, well-characterized cohorts and report diagnostic metrics with confidence intervals, enabling a more realistic evaluation of translational potential. Systematic comparisons of circRNA measurements across sample types within the same patient cohorts are needed to establish comparability and guide sample type selection. In parallel, direct comparisons between tissue and matched plasma samples are essential to determine whether tissue-derived subtype signatures can be reliably detected in circulation [157].

Methodological standardization also remains a critical priority. The absence of standardized preanalytical protocols, covering blood collection tube type, processing time, centrifugation conditions, and storage temperature, can markedly affect circRNA yield and integrity. Similarly, no consensus currently exists regarding optimal normalization methods for plasma circRNA quantification. Some studies rely on endogenous reference genes such as glyceraldehyde-3-phosphate dehydrogenase (GAPDH) or U6 small nuclear RNA (U6), whereas others employ synthetic spike-in controls [158]. Systematic evaluation of different approaches is needed to establish standardized protocols. Rigorous assay validation is equally essential; adherence to established guidelines such as MIQE (Minimum Information for Publication of Quantitative Real-Time PCR Experiments) and MISEV (Minimal Information for Studies of Extracellular Vesicles) would improve the reliability and transparency of circRNA biomarker studies [159].

Encouragingly, the translational potential of these biomarkers is already being explored in clinical settings. An ongoing trial (NCT05771337) is investigating the diagnostic value of serum circRNAs in BC patients [17]. Beyond diagnostic applications, the success of the phase III KEYNOTE-522 trial has established chemoimmunotherapy as the standard of care for high-risk early-stage TNBC, with pembrolizumab plus chemotherapy achieving a 7-year event-free survival rate of 78.3% versus 69.8% with chemotherapy alone [160]. This paradigm shift in TNBC treatment underscores the pressing need for predictive biomarkers such as circRNAs that can identify patients most likely to benefit from immunotherapy and guide personalized treatment strategies.

### 9.4 Strategic Framework for Clinical Adoption

To facilitate the translation of circRNA biomarkers from exploratory findings into clinical practice, they should be embedded within retrospective-prospective clinical trial frameworks. This strategy entails retrospective analysis of biospecimens from completed trials to generate preliminary evidence, prospective validation in independent cohorts to confirm diagnostic or prognostic performance, and ultimate integration into ongoing studies to evaluate their incremental value over existing biomarkers. Realizing this vision requires coordinated action among academic institutions, clinical trial networks, and diagnostic companies to formulate consensus guidelines, unify detection protocols, and accelerate the clinical adoption of circRNA biomarkers [161].

## 10 Conclusion

CircRNAs have attracted considerable interest as non-invasive biomarkers for BC, with potential applications in early detection, prognosis, and treatment monitoring, owing to their high stability in circulation and tissue-restricted expression patterns. In this review, we have synthesized current evidence on plasma circRNAs as diagnostic, prognostic, chemoresistance-related, and recurrence-related biomarkers, while also discussing detection considerations, molecular mechanisms, and the challenges that remain for clinical translation. Despite their promise, several obstacles persist, including the absence of standardized protocols, a lack of clinically validated cut-off values, and the limited specificity of circRNAs, which are not cancer-exclusive and can be altered in multiple pathological conditions. Moreover, although tissue-based studies have identified subtype-specific circRNA signatures, their applicability to plasma-based biomarkers remains largely unsubstantiated, highlighting the need to bridge the tissue-to-plasma gap. Future progress will likely depend on multi-marker panels or the integration of circRNAs with other circulating analytes, such as ctDNA and exosomal proteins, to achieve the specificity required for clinical decision-making. With continued research, technological refinement, and rigorous multi-center validation, circRNA-based liquid biopsy may become an integral component of precision oncology in BC management.

## Data Availability

Not applicable.

## Abbreviations

ABCB10	ATP-binding cassette subfamily B member 10
ABUS	Automated breast ultrasound
ADAR	Adenosine deaminase acting on RNA
AGO2	Argonaute2
AKT	Protein kinase B
Akt1	RAC-alpha serine/threonine-protein kinase
ALN	Axillary lymph nodes
Alu	Alu sequence
ANKRD46	Ankyrin repeat domain 46
ANXA2	Annexin A2
APC	Adenomatous polyposis coli
Aptasensors	Aptamer-based biosensors
ASO	Anti-sense oligonucleotide
ASOs	Antisense oligonucleotides
ATG7	Autophagy-related gene 7
ATG13	Autophagy-related protein 13
AUC	Area under the curve
BCBM1	breast cancer brain metastasis 1
BCL11A	B-cell lymphoma/leukemia 11A
BETi	Bromodomain and extra-terminal domain inhibitors
Biosensors	Biosensor-based detection platforms
BISC	BETi sensitizing circRNA
BRD4	Bromodomain-containing protein 4
BSJ	Back-splice junction
BSS	Back-splice site
CA15-3	Cancer antigen 15-3
CA 27.29	Cancer antigen 27.29
CAFs	Cancer-associated fibroblasts
CAR	Chimeric antigen receptor
CAR-NK	Chimeric antigen receptor-natural killer
Cas9	CRISPR-associated protein 9
CCL20	Chemokine (C-C motif) ligand 20
CCND1	Cyclin D1
CCT2	Chaperonin containing TCP1 subunit 2
CDK4	Cyclin-dependent kinase 4;
CDK6	Cyclin-dependent kinase 6
CDYL	Chromodomain Y-like
CDYL2	Chromodomain Y like 2
CEA	Carcinoembryonic antigen
ceRNA	Competing endogenous RNA
CircCDR1as	CircRNA CDR1as
circRNAs	Circular RNAs
CircNet	An updated database for exploring circular RNA regulatory networks in cancers
Cirscan	CIRcular RNA sponge CANdidates
CiRNAs	Circular intronic RNAs
CKI	Casein kinase I
CLASP1	Cytoplasmic linker associated protein 1
CLIP-seq	Cross-linking and immunoprecipitation sequencing
C-myc	Cellular myelocytomatosis oncogene (or MYC proto-oncogene)
CRISPR	Clustered regularly interspaced short palindromic repeats
CRISPR-Cas13d	Clustered regularly interspaced short palindromic repeats-CRISPR associated protein 13d
CSF2	Colony stimulating factor 2
CTCs	Circulating tumor cells
ctDNA	Circulating tumor DNA
C-TNK2-487aa	C-terminal TNK2-encoded 487-amino acid protein
CXCL10	C-X-C motif chemokine ligand 10
CXCR4	C-X-C chemokine receptor type 4
CYR61	Cysteine-rich angiogenic inducer 61
ddPCR	Droplet digital polymerase chain reaction
DFS	Disease-free survival
DSS	Disease-specific survival
DUSP7	Dual specificity phosphatase 7
DVL	Dishevelled segment polarity protein
EcircRNAs	Exonic circular RNAs
EDTA	Ethylenediaminetetraacetic acid
EGFR	Epidermal growth factor receptor
EIciRNAs	Exon-intron circular RNAs
EMT	Epithelial-mesenchymal transition
ENCORI	Encyclopedia of RNA Interactomes
EPSTI1	Epithelial stromal interaction 1
ER	Estrogen receptor
ERBB2	Erb-B2 receptor tyrosine kinase 2
ERBB3	Erb-B2 receptor tyrosine kinase 3
ERK	Extracellular signal-regulated kinase
ETS1	E26 transformation-specific sequence 1
EXPAR	Exponential amplification reaction
FA K	Focal adhesion kinase
FAF1	Fas-associated factor 1
FBXW7	F-Box/WD repeat-containing protein 7
FGFR4	Fibroblast growth factor receptor 4
FOXK2	Forkhead box protein K2
FUS	Fused in sarcoma
FZ	Frizzled
GAPDH	Glyceraldehyde-3-phosphate dehydrogenase
GDP	Guanosine diphosphate
GGNBP2	Gametogenetin binding protein 2
GINS4	GINS complex subunit 4
GLI1	Glioma-associated oncogene homolog 1
gp130	Glycoprotein 130
GPNMB	Glycoprotein non-metastatic melanoma protein B
GRB7	Growth factor receptor bound protein 7
GSK3	Glycogen synthase kinase 3
GTP	Guanosine triphosphate
HER2	Human epidermal growth factor receptor 2
HHUS	Handheld ultrasound
HIPK3	Homeodomain interacting protein kinase 3
HK2	Hexokinase 2
HMGA1	High mobility group A1
HNRNPL	Heterogeneous nuclear ribonucleoprotein L
HOXA1	Homeobox A1
hsa	Homo sapiens
ICSs	Intronic complementary sequences
IGF2BP2	Insulin-like growth factor 2 mRNA-binding protein 2
IRES	Internal ribosome entry sites
ISL	Isoliquiritigenin
IL-6R	Interleukin 6 receptor
JAK	Janus kinase
Ki-67	Proliferation marker protein Ki-67
LAMP	Loop-mediated isothermal amplification
LNPs	Lipid Nanoparticles
LRP6	Low-density lipoprotein receptor-related protein 6
M2 TAMs	M2 tumor-associated macrophages
m^6^A	N^6^-methyladenosine
MAPK	Mitogen-activated protein kinase
MAPKK	Mitogen-activated protein kinase kinase
MAPKKK	Mitogen-activated protein kinase kinase kinase
MAVS	Mitochondrial antiviral signaling
MBL	Muscleblind
MBNL1	Muscleblind-like splicing regulator 1
MEK	Mitogen-activated protein kinase kinase
MIQE	Minimum information for publication of quantitative real-time PCR experiments
miRNA	microRNA
MISEV	Minimal information for studies of extracellular vesicles
MMP1	Matrix metalloproteinase 1
MREs	MiRNA response elements
mRNA	messenger RNA
MRI	Magnetic resonance imaging
mTOR	Mechanistic target of rapamycin
NAC	Neoadjuvant chemotherapy
Nanopore	Nanopore sequencing
NanoString	NanoString nCounter analysis system
NF1	Neurofibromin 1
NK	Natural killer
Northern blotting	Northern blot hybridization
NOTCH1	Notch receptor 1
NR	Not reported
NTRK3	Neurotrophic receptor tyrosine kinase 3
OS	Overall survival
p53	Tumor protein 53
PacBio	Pacific biosciences single-molecule real-time sequencing
panCAR	pan-chimeric antigen receptor
PARPBP	PARP1 binding protein
PCR	Polymerase chain reaction
pCR	pathological complete response
PD-1	Programmed cell death protein 1
PDK4	Pyruvate dehydrogenase kinase 4
PFS	Progression-free survival
PGR	Progesterone receptor
PI3K	Phosphoinositide 3-kinase
PIK3R2	Phosphoinositide-3-kinase regulatory subunit 2
PIP2	Phosphatidylinositol 4,5-bisphosphate
PIP3	Phosphatidylinositol (3,4,5)-trisphosphate
PLK1	Polo-like kinase 1
PR	Progesterone receptor
p-RB-E2F	Phosphorylated retinoblastoma protein-E2F transcription factor
pre-mRNAs	Precursor messenger RNAs
PRMT5	Protein arginine methyltransferase 5
PSMA1	Proteasome subunit alpha type 1
PTEN	Phosphatase and tensin homolog
PTK2	Protein tyrosine kinase 2
PVT1	Plasmacytoma variant translocation 1
QKI	Quaking
qPCR	Quantitative real-time polymerase chain reaction
RAF	The rapidly accelerated fibrosarcoma
RAS	Rat sarcoma
RASSF2	Ras association domain-containing protein 2
RBPs	RNA-binding proteins
RCA	Rolling circle amplification
RISC	RNA-induced silencing complex
RHOT1	Ras homolog family member T1
RNAi	RNA interference
RNA Pol II	RNA polymerase II
RNA-seq	RNA sequencing
RNA-Seq (long-read)	Long-read RNA sequencing
RNA-Seq (short-read)	Short-read RNA sequencing
RNase R + qPCR	Ribonuclease R treatment combined with quantitative polymerase chain reaction
ROBO4	Roundabout guidance receptor 4
RPAP2	RNA polymerase II associated protein 2
RT-qPCR	Reverse-transcription quantitative real-time polymerase chain reaction
SCAP	SREBP cleavage-activating protein
SERS	Surface-enhanced Raman scattering
SFRP1	Secreted frizzled-related protein 1
shRNA	Short hairpin RNA
siRNA	Small interfering RNA
SNAIL	Snail family transcriptional repressor 1
SOCS3	Suppressor of cytokine signaling 3
SOS1	Son of sevenless 1
SOX2	SRY-box transcription factor 2
SRCAP	Snf2-related CREBBP activator protein
SRSF1	Serine and arginine rich splicing factor 1
STAT3	Signal transducer and activator of transcription 3
STIL	SCL-interrupting locus protein
TAMs	Tumor-associated macrophages
TCF7L2	Transcription factor 7 like 2
TFAP2C	Transcription factor AP-2 gamma
TFEB	Transcription factor EB
THP	Pirarubicin
TNBC	Triple-negative breast cancer
TNK2	Tyrosine kinase non receptor 2
TPR	Translocated promoter region
U1 snRNP	U1 small nuclear ribonucleoprotein
U6	U6 small nuclear RNA
ULK1	Unc-51 like autophagy activating kinase 1
US	Ultrasound
UTR	Untranslated region
Wnt	Wingless-related integration site
WWC3	WW and C2 domain containing 3
YWHAZ	Tyrosine 3-monooxygenase/tryptophan 5-monooxygenase activation protein zeta
ZNF217	Zinc finger protein 217
ZNF704	Zinc finger protein 704
